# Antiviral treatment in adult patients hospitalized for influenza: study protocol for a multi-center, randomized, placebo-controlled trial on the efficacy of baloxavir marboxil to reduce time to clinical improvement and the risk for severe complications (the INFLUENT trial)

**DOI:** 10.1186/s13063-025-09248-0

**Published:** 2025-11-24

**Authors:** Krisztina Hosszu-Fellous, Antoine Poncet, Ana Rita Goncalves Cabecinhas, Manuel Schibler, Benjamin Meyer, Virginie Prendki, Angela Huttner, Sebastian Carballo, Mathias Pouillon, Ksenija Slankamenac, Pierre-Alexandre Bart, Enos Bernasconi, Oriol Manuel, Matteo Mombelli, Nicolas J. Mueller, Laurent Kaiser, Pauline Vetter

**Affiliations:** 1 Center for Emerging Viral Diseases, Geneva University Hospitals, 4, Rue Gabrielle-Perret Gentil, Geneva, 1205 Switzerland; 2 Clinical Research Centre, Faculty of Medicine, University of Geneva, Geneva, Switzerland; 3 Department of Diagnostics, Laboratory of Virology, Geneva University Hospitals, Geneva, Switzerland; 4 Department of Pathology and Immunology, Faculty of Medicine, Center of Vaccinology, University of Geneva, Geneva, Switzerland; 5 Department of Medical Specialties, Division of Infectious Diseases, Geneva University Hospitals, Geneva, Switzerland; 6 Department of Rehabilitation and Geriatrics, Division of Internal Medicine for the Aged, Geneva University Hospitals, Geneva, Switzerland; 7 Division of Internal Medicine, Department of Medical Specialties, Geneva University Hospitals, Geneva, Switzerland; 8 Emergency Department, Geneva University Hospitals, Geneva, Switzerland; 9 Emergency Department, University Hospital Zurich, Zurich, Switzerland; 10 Department of Internal Medicine, Lausanne University Hospital, Lausanne University Medical School, Lausanne, Switzerland; 11 Division of Infectious Diseases, Lugano Regional Hospital EOC, University of Geneva and University of Southern Switzerland, Lugano, Switzerland; 12 Department of Infectious Diseases and Center for Organ Transplantation, Lausanne University Hospital, Lausanne, Switzerland; 13 Department of Internal Medicine, Mendrisio Regional Hospital EOC, Mendrisio, Switzerland; 14 Department of Infectious Diseases and Hospital Epidemiology, University Hospital Zurich, Zurich, Switzerland

**Keywords:** Influenza, Flu, Antiviral, Treatment, Hospitalization, Adult, Randomized, Clinical trial

## Abstract

**Background:**

Seasonal influenza virus leads to more than half a million deaths each year worldwide. Due to its capacity to evolve, it is considered the most likely pathogen to cause a future pandemic. Antiviral treatment options are currently limited, with the most widely used drugs being neuraminidase inhibitors and the cap-dependent endonuclease inhibitor baloxavir marboxil. In adult hospitalized patients, due to the lack of placebo-controlled trials, current available evidence on antiviral treatment benefits is essentially based on observational studies. A placebo-controlled clinical trial is needed to fill this knowledge gap.

**Methods:**

This is an investigator-initiated, randomized, triple-blind, placebo-controlled, superiority, multi-center trial to assess the clinical efficacy of single-dose baloxavir in decreasing time to clinical improvement in adult immunocompetent patients hospitalized with influenza. Patients (*n* = 484) with confirmed, severe infection (NEWS2 score ≥ 4) will be recruited over three influenza seasons in four large Swiss hospitals. Primary outcome: time to clinical improvement (in hours), calculated from treatment administration until NEWS2 score ≤ 2 maintained for 24 h or until hospital discharge, whichever comes first. The primary outcome is calculated in all patients independently of the duration of symptoms at treatment administration, as well as in participants treated early (<72 h) post onset of symptoms. The main secondary outcomes are the risk of serious influenza complications, length of hospitalization, and difference in viral load at D3 post-treatment administration.

**Discussion:**

This trial’s results, whether positive or negative, will impact clinical guidance. If baloxavir’s clinical benefit is demonstrated, a single-dose pill would be the easiest implementable treatment option in case of large seasonal outbreaks or a new influenza pandemic. If a clear treatment benefit is not shown, antiviral treatment administration in the hospitalized patient population could be reconsidered to prevent unnecessary medication, lower the risk of resistance development linked to treatment overuse, and ultimately save unnecessary treatment-related expenses.

**Trial registration:**

ClinicalTrials.gov NCT06653569. Registered on October 22, 2024, https://clinicaltrials.gov/study/NCT06653569?term=NCT06653569&rank=1.

**Supplementary Information:**

The online version contains supplementary material available at 10.1186/s13063-025-09248-0.

## Administrative information

**Table:** 

Title {1}	A Swiss multi-center, randomized, placebo-controlled trial on the efficacy of baloxavir marboxil to reduce time to clinical improvement in adult patients hospitalized for influenza, the INFLUENT trial (INpatients inFLUENza Treatment)
Trial registration {2a and 2b}	ClinicalTrials.gov NCT06653569 Swiss National Clinical trial Portal SNCTP000006168 BASEC: 024-01535
Protocol version {3}	Version 2.0, October 29, 2024
Funding {4}	Swiss National Science Foundation (Grant number 22 170) Geneva University Hospitals (Fonds institutionnel du Service des maladies infectieuses des Hôpitaux Universitaires de Genève)
Author details {5a}	Pauline Vetter, Krisztina Hosszu-Fellous Center for Emerging Viral Diseases, Geneva University Hospitals, Geneva, Switzerland Antoine Poncet Clinical Research Centre, Faculty of Medicine, University of Geneva, Geneva, Switzerland Manuel Schibler, Ana Rita Goncalves Cabecinhas Laboratory of Virology, Department of Diagnostics, Geneva University Hospitals, Geneva, Switzerland Virginie Prendki Department of Medical Specialties, Division of Infectious Diseases, Geneva University Hospitals, Geneva, Switzerland Division of Internal Medicine for the Aged, Department of Rehabilitation and Geriatrics, Geneva University Hospitals, Geneva, Switzerland Angela Huttner, Laurent Kaiser Department of Medical Specialties, Division of Infectious Diseases, Geneva University Hospitals, Geneva, Switzerland Sebastian Carballo Department of Medical Specialties, Division of Internal Medicine, Geneva University Hospitals, Geneva, Switzerland Mathias Pouillon Emergency Department, Geneva University Hospitals, Geneva, Switzerland Ksenija Slankamenac Emergency Department, University Hospital Zurich, Zurich, Switzerland Enos Bernasconi Division of Infectious Diseases, Lugano Regional Hospital EOC, University of Geneva and University of Southern Switzerland, Lugano, Switzerland Oriol Manuel Department of Infectious Diseases and Center for Organ Transplantation, Lausanne University Hospital Pierre-Alexandre Bart Department of Internal Medicine, Lausanne University Hospital; Lausanne University Medical School, Lausanne, Switzerland Matteo Mombelli Department of Internal Medicine, Locarno Regional Hospital EOC, Locarno, Switzerland Nicolas J Mueller Department of Infectious Diseases and Hospital Epidemiology, University Hospital Zurich, Zurich, Switzerland
Name and contact information for the trial sponsor {5b}	Sponsor: Geneva University Hospitals, 4, rue Gabrielle-Perret Gentil, 1205 Geneva, Switzerland Sponsor representative: Dr. Pauline Vetter, Geneva Center for Emerging Viral Diseases, Division of Infectious Diseases and Laboratory of Virology, Geneva University Hospitals, 4, rue Gabrielle-Perret Gentil, 1205 Geneva, Switzerland +41 79 55 39 761, Pauline.vetter@hug.ch
Role of sponsor {5c}	Funders have no role in study design, collection, management, analysis and interpretation of the data, writing of the report, and decision to submit the report for publication. The sponsor by its representative takes the responsibility to initiate, manage, and find funding for this trial, as well as to analyze and interpret the data, write the report, and decide to submit the report for publication

## Introduction

### Background and rationale {6a}

Each year, influenza causes a severe disease burden worldwide. Every winter in Switzerland, several thousands of patients require hospitalization due to complicated influenza, with hundreds of deaths [1]. Treatment strategies contributing to a shorter hospital stay as well as to a lower risk of complications have major public health importance.

In addition, due to its immense capacity to evolve, influenza is one of the leading causes of human pandemics of respiratory origin [2]. The avian influenza A(H5N1) strains causing the epizootic ongoing since 2020 are not yet adapted to humans. However, each new spillover increases the risk of mutations allowing adaptation to the mammalian host [3]. Determining the best possible use of antiviral treatment resources will be crucial in case of a new pandemic.

The most frequently used antiviral drugs to treat influenza in hospitalized patients are neuraminidase inhibitors (NAI) including oseltamivir and the cap-dependent endonuclease inhibitor baloxavir marboxil (baloxavir from thereof). For uncomplicated cases in high-risk outpatients, a 16-to-25-h reduction in symptom duration was observed when oseltamivir was administered within 48 h post onset of symptoms (POS) [4–6].

Importantly, no placebo-controlled trial on influenza antivirals in adult hospitalized patients has ever been published [5]. Current available evidence is based on a series of observational studies and their systematic reviews. Most of them identify treatment timing as an important factor modulating potential efficacy. In two retrospective analyses of patient data during the influenza A(H1N1) pandemic, early (≤48 h POS) oseltamivir treatment was associated with lower mortality risk (aOR 0.50, 95% CI 0.37, 0.67) as well as with shorter in-hospital stay (−1.19 day, IQR −0.85, −1.55) [7, 8]. Late oseltamivir treatment benefits (>48 h POS) were only shown in patients requiring intensive care upon admission (mortality risk OR 0.65, 95% CI 0.46, 0.93) [7]. Some of these findings have been criticized for high risk of survival and selection bias, confounding by disease severity, as well as for industry funding [4, 9]. A recently published large retrospective study demonstrated an 18% decrease in 30-day mortality risk associated with oseltamivir treatment in patients infected with influenza A, but not with influenza B. The association remained significant when antiviral was administered ≥48 h after hospitalization [10]. Another study showed an association between prompt antiviral treatment at hospitalization and 30-day mortality risk decrease when compared to delayed treatment administration (treatment on day 1: aOR 1.14, 95% CI 1.01, 1.27; between days 2 and 5: aOR 1.40, 95% CI 1.17, 1.66) [11].

On the other hand, a recent systematic review and network meta-analysis of randomized controlled trials in children and adults, comparing NAI treatment to placebo (in children)/to standard of care or to each other (in adults), highlighted the uncertainty of the efficacy of NAI to lower the risk of mortality (risk difference: −18 to +4/1000) or admission to ICU, and concluded that treatment may shorten the duration of hospitalization (for oseltamivir: −1.63 days (95% CI: −2.81 to −0.45) (low level of evidence)) [12, 13]. In hospitalized patients, there is only one retrospective observational study assessing the clinical benefits of baloxavir, showing a significant 30-day all-cause mortality reduction in the baloxavir group compared to oseltamivir when treatment was administered within 48 h POS [14]. Combination antiviral therapies (baloxavir and NAI) could in theory increase clinical effectiveness via the cumulation of antiviral potency (additive or synergistic effects) as well as by reducing the risk of antiviral resistance [15]. However, one phase III multi-center randomized clinical trial in hospitalized adult patients failed to show a benefit in time to symptom alleviation when compared to NAI monotherapy [16]. Due to the lack of strong evidence, physicians remain divided over whether patients hospitalized with complicated influenza would benefit from antiviral administration and, if yes, for how long after symptom onset.

As the equipoise exists regarding treatment efficacy, a placebo-controlled trial is needed to fill in this knowledge gap.

### Objectives {7}

Primary objective: To measure the clinical efficacy of single-dose baloxavir in shortening the time to clinical improvement compared to placebo in adult immunocompetent patients hospitalized for influenza irrespective of the duration of symptoms.

Co-primary objective: To measure the clinical efficacy of single-dose baloxavir in shortening the time to clinical improvement compared to placebo in the subgroup of adult immunocompetent patients hospitalized for influenza and treated less than 72 h POS. Secondary objectives: To assess baloxavir’s efficacy in decreasing the risk for and the duration of oxygen therapy related to the influenza episode, as well as its efficacy in preventing serious influenza-related complications; to measure its effects on viral-load kinetics including duration of infectiousness.

### Trial design {8}

This is an investigator-initiated, randomized, triple-blind, placebo-controlled, parallel group, superiority, multi-center trial with an allocation ratio of 1:1.

## Methods: participants, interventions and outcomes

### Study setting {9}

The trial is implemented in four large Swiss hospitals: Geneva University Hospitals; University Hospital Zurich; University Hospital Lausanne; Locarno and Lugano Regional Hospitals.

### Eligibility criteria {10}

#### Inclusion criteria

- Age ≥ 18 years
- Written informed consent provided by the patient or by patient’s family member
- Positive reverse transcriptase-polymerase chain reaction (RT-PCR) for influenza A and/or B on a respiratory tract specimen
- Participant requiring hospitalization
- National Early Warning Score 2 (NEWS2) of ≥4 at planned randomization

#### Exclusion criteria

- Ongoing pregnancy or breastfeeding
- Known contraindication to baloxavir or to placebo
- Participant weighting <40 kg
- Prior treatment with influenza antiviral for the current influenza episode (NAI therapy for >24 h at the time of randomization or one dose of baloxavir)
- Immunosuppression defined as (1) cancer treatment with significant negative effect on the immune system as determined by study investigator; (2) immunosuppressive therapy (treatment comprising ≥20 mg/day prednisone or equivalent when administered for ≥2 weeks, biological therapies, steroid sparing drugs); (3) HIV infection if CD4+T cell count <500/µL; (4) organ or stem-cell transplantation; and (5) patients awaiting transplant
- Severe underlying respiratory comorbidity requiring long-term oxygen therapy at home
- Severe disease requiring ICU care, need for high flow oxygen or non-invasive ventilation at the time of randomization
- Severe hepatic insufficiency or any other severe medical condition when participation in the study puts the patient at risk according to the investigator’s judgment
- Prior inclusion in this study during a previous influenza season
- Inclusion in another interventional study with an investigational drug 30 days before or planed within 30 days after inclusion in the study
- Inability to consent or patient representative unable to consent

### Who will take informed consent? {26a}

Hospitalized patients with a positive RT-PCR result for influenza and requiring hospitalization are going to be screened for study inclusion and exclusion criteria. In case of study eligibility, patients will be approached by the study staff. The recruiting trial physician or nurse will explain to each patient or in case of a patient incapable of judgment to his/her legal representative the nature of the study. Those who sign the informed consent (the legal representative in case of a patient is incapable of judgment) will be included in the study and will be randomized.

### Additional consent provisions for collection and use of participant data and biological specimens {26b}

Participants will be asked to sign another separate consent for inclusion in the corresponding INFLUENT biobank resulting in further use of their clinical data and biological specimen in case of future studies. The consent also includes additional serum sampling for the biobank on the day of randomization, as well as on day 7 post-treatment administration.

## Interventions

### Explanation for the choice of comparators {6b}

Explanation for the choice of the study treatment: We chose baloxavir over oseltamivir to study the benefits of antiviral treatment: (1) to add additional treatment options to the existing choice of neuraminidase inhibitors in the hospitalized patient population, given the high mutational potential of influenza viruses and (2) due to baloxavir’s ease of use and its excellent tolerability in clinical studies [13, 16].

Explanation for the choice of the comparator: We believe that comparing baloxavir therapy to placebo in our patient population is ethical: (1) given the persisting equipoise regarding the benefits of antiviral treatment in patients hospitalized for influenza; (2) treatment should be compared to placebo in order to identify the patient population that stands to gain the most from antivirals (to further increase treatment accessibility, curtail unnecessary expenses, and mitigate the risk of emergence of resistance and adverse effects). We adapted inclusion and exclusion criteria to exclude patients who might benefit from antiviral treatment, such as the critically ill and the immunocompromised patients.

### Intervention description {11a}

A total of 484 participants will be included, with 242 randomized in each arm. In the treatment arm, participants will receive baloxavir marboxil (Xofluza®) according to their body weight (<80 kg: 1 tablet of 40 mg PO as a single dose, ≥80 kg: 2 tablets of 40 mg PO as a single dose). Participants randomized to the placebo arm will receive one single dose of placebo. The number of placebo capsules will be adapted to the patient’s weight (<80 kg: 1 capsule, ≥80 kg: 2 capsules).

### Criteria for discontinuing or modifying allocated interventions {11b}

As the intervention consists of a single dose of study treatment, discontinuation of the intervention is not applicable here. All participants will be free at any time and for any reason to withdraw from the study without having to give any explanation.

### Strategies to improve adherence to interventions {11c}

As the intervention consists of a single-dose study treatment and the primary endpoint is based on inpatient clinical data or the length of hospitalization, we do not expect any loss to follow-up. To ensure the feasibility of the 3-month telephone follow-up, for each inclusion the contact of a relative will be noted. In addition, the patient’s general practitioner may be contacted in case the participant or his/her relative is not able to provide adequate information at the 3-month follow-up visit. The study team can also review patient electronic health records to retrieve information about potential influenza complications and their treatment up to 90 days post-treatment administration.

### Relevant concomitant care permitted or prohibited during the trial {11d}

All concomitant medications are allowed except other investigational medicinal products (IMP) up to 30 days prior and after randomization. “As the secondary objectives measure baloxavir’s efficacy in decreasing the risk for severe influenza complications up to 90 days post-treatment administration, the use of other approved or experimental influenza antivirals after randomization and for 90 days post-treatment administration will be considered a deviation of the protocol.”

### Provisions for post-trial care {30}

In case of a serious adverse event unrelated to the natural course of the current influenza episode or in case of SAR and SUSAR, participants will be followed until event resolution. An insurance covering the study activities has been contracted through the sponsor’s institution, the Geneva University Hospitals.

### Outcomes {12}

#### Primary outcome

The primary outcome is time to clinical improvement (in hours), calculated from treatment administration until the measurement of a NEWS2 score ≤ 2 maintained for 24 h, or until hospital discharge, whichever comes first.

#### Secondary clinical outcomes

1. Clinical status severity score (from 1 to 6) on day 7 post-treatment administration
2. Duration of hospitalization post-treatment administration (in hours)
3. Duration of O_2_ supplementation post-treatment administration (in hours)
4. In-hospital clinical failure, defined as death or ICU or intermediate care unit (ICMU) admission during hospitalization or up to 30 days post-treatment administration in case of prolonged hospital stay
5. Mortality at D90
6. Influenza-related complications occurring between D0 and D90, diagnosed by the treating physician (composite outcome defined as pneumonia, sepsis, acute lung injury or acute respiratory distress syndrome (ARDS), encephalitis/encephalopathy, myo- or pericarditis, otitis, sinusitis as well as any cardiovascular event (cardiac decompensation, myocardial infarction, or stroke))
7. Number of antibiotic days (AD) prescribed during hospitalization or up to 30 days post-treatment administration in case of longer hospital stay
8. Quality of life at day 90 post-treatment administration. Questionnaire type: EQ-5D-5L

#### Secondary virology outcomes

1. Detection of RNA in respiratory sample on day 3 post-treatment administration
2. Infectious viral load in respiratory sample at day 3 post-treatment administration

### Participant timeline {13}

Patients will be followed up during hospitalization; data on clinical symptom severity, as well as flu complications (exacerbation of underlying disease, bacterial superinfection, need for ICU admission, death), antibiotic use, and duration of hospitalization will be collected. Nasopharyngeal swab for virological endpoints will be taken on day 0 and day 3 following treatment administration. After discharge, one phone follow-up visit is planned 90 days post-treatment administration (Table 1).

**Table 1:** Patient timeline, screening, inclusion, treatment and follow-up schedule

**Study visit**	**1**	**0**	**2**	**3.1**	**3.2**	**3.3**	**3.4**	**3.5**	**3.6**	**3.7**	**3.8**	**3.9**	**3.10**	**4**	**5**
	**Screening**	**Consent**	**Allocation**	**In-hospital follow-up**											
**Timepoint**	Day −2 to D0	D0	D0	D1	D2	D3	D4	D5	D6	D7	D8	D9	…	Discharge day	D90
**Window (in days)**															±10
**Informed consent**		x													
**Inclusion and exclusion criteria**	x														
**Flu symptoms history**			x												
**Medical history**			x												
**Demographics**			x												
**Quality of life questionnaire**			x												x
**NEWS2 score**	x		x	1/8 h until discharge (a)											
**Clinical symptoms severity evaluation**			x	1/D until discharge											
**Influenza complications evaluation**			x	1/D until discharge											x
**6-point ordinal scale**										x					
Serious adverse events*			x	During hospitalization											x
**Laboratory results collection**			x^b,c^	Once between D2 and D4^b,c^											
**RT-PCR NP swab**			x^d^			x^d^									
**Viral culture**			x^e^			x^e^									
**Viral type and subtype**			x												
**Screening for resistance-associated mutations**			x^f^			x^f^									
**Biobaking: serum sampling**			(x)							(x)					

a: 1/8 h up to 10 days. If hospitalization is longer and the primary endpoint is still not reached, decrease NEWS2 score measurements to 1/day up to discharge. b: only if requested by the treating physician. c: only CRP and white blood cell count will be collected and only twice during hospitalization, first at D0 and second if available between D2 and D4. d: ±1 day. e: only if RT-PCR positive with CT value compatible with viral growth (CT value < 30). f: depending on budget availability. (x): 6 ml, only at HUG, only consenting participants accepting blood sample collection for future research, timepoint of collection ± 1D. *: All SAE will be collected and reported in the annual safety report. SARS and SUSAR will be reported timely to the regulatory authorities

### Sample size {14}

Our hypothesis is that baloxavir treatment in adult, immunocompetent patients hospitalized for influenza will shorten the time to clinical improvement by at least 36 h compared to placebo. This 36-h difference was chosen as the minimal time difference considered to have a meaningful impact on clinical care. The median time to clinical improvement, in patients who received antivirals in a large clinical study, was about 4 days (97.5 h) [16]. The addition of the 36-h difference equals a time to clinical improvement of 5.5 days in the placebo group.

Two sample size calculations were performed. Sample size calculation no. 1 concerns all patients hospitalized for influenza (regardless of symptom duration at treatment administration): under the constant and proportional hazard assumption, median times to clinical improvement of 5.5 days in the placebo group and 4 days in the baloxavir group correspond to a hazard ratio of 1.375. With a follow-up period of 30 days, we calculated that a total number of 432 participants (216 per arm) would provide the trial with at least 90% power to detect such a baloxavir effect on time to clinical improvement at a two-sided significance level of 0.05.

Sample size calculation no. 2 concerns the subgroup of patients hospitalized for influenza with symptoms evolving for less than 72 h: under similar hypothesis (median times to clinical improvement of 5.5 days in the placebo group and 4 days in the baloxavir group, corresponding to a hazard ratio of 1.375 under the constant and proportional hazard assumption, and a follow-up period of 30 days), we calculated that a sample of 161 patients per arm would provide 80% power to detect a baloxavir effect in a subgroup analysis, at a two-sided significance level of 0.05. Assuming two out of three patients will present symptoms evolving for less than 72 h (ratio of 2:1), the study will need to include a total of 484 patients. With that sample size, the study will reach a power of 93% in the general population of hospitalized patients.

### Recruitment {15}

We aim to recruit 484 participants in total (242 in each arm). We estimate that we will be able to reach this number in 3 influenza seasons. Should we be unable to recruit the necessary number of participants each year, enrollment could be prolonged for a fourth year. In case of a low number of influenza patients during future seasons, seasonal recruitment makes it relatively easy to organize the opening of new centers during spring/summer.

## Assignment of interventions: allocation

### Sequence generation {16a}

Patient randomization was performed by using randomization blocks of varying sizes. Randomization is stratified only by study site. One specific randomization list was prepared for each study site before the first influenza season started (in November 2024). The randomization lists cover all three influenza seasons and are 50% longer than the number of participants intended to be included. The pharmacist prepares treatment and placebo kits in advance, each having a randomization number. After study inclusion, kits are allocated to each patient following the order of inclusion.

### Concealment mechanism {16b}

The randomization list has been prepared by an external person to the study and has been only made accessible to the pharmacist of the Geneva University Hospitals who prepares the medication kits. Thus, the treatment allocation is concealed from patients, physicians, and trial statistician and all other involved study personal.

### Implementation {16c}

Randomization list generation was done by an expert in clinical trials who is not part of the study team. Dedicated nurses and study physicians oversee patient enrollment. Assignment to the intervention is done at randomization by the study nurse, who takes the next available kit prepared in advance by the Geneva University Hospitals pharmacist.

## Assignment of interventions: blinding

### Who is blinded {17a}

The study is triple-blind, with study participants, study nurse/physician, treating physician, and data analyst all blinded to the patients’ intervention group. Emergency unblinding is possible 24/7.

Baloxavir and placebo packaging are similar, ensuring the double blinding.

As mentioned above, the person in charge of creating the randomization list and the physician keeping a copy of the randomization list are not part of the study.

All unblinding events will be recorded as a deviation of the protocol in the TMF.

### Procedure for unblinding if needed {17b}

Unblinding is possible:

- In case of a life-threatening event suspected to be related to therapy (SAR or SUSAR).
- In case this information is needed by the treating physician to provide the best possible care for the patient.

The pharmacy at the Geneva University Hospitals keeps a copy of the randomization list in case of necessity for emergency unblinding, which is possible 24/7 by the pharmacist on duty.

As the treatment is only administered in a single dose, and does not preclude the administration of another antiviral active against influenza viruses, the need for emergency unblinding is very low.

## Data collection and management

### Plans for assessment and collection of outcomes {18a}

Primary outcome: We use the NEWS2 scoring system as a reliable and easily implementable method to evaluate disease severity at inclusion and to measure improvement in clinical status during follow-up. The primary outcome (NEWS2 score or hospital discharge) is recorded in the CRF once a day by dedicated study nurses. Components of the NEWS2 score are part of regular clinical management and therefore recorded by the team in charge of the patient during hospitalization. Death or ICU admission is not considered as discharge in the context of the primary endpoint. In case the patient is transferred to another hospital, it is considered as discharge regarding the primary endpoint. Transfers from the four participating university centers most often occur to rehabilitation hospitals, indicating that the patient no longer requires acute care for the influenza episode and is thus considered discharged.

To evaluate change in clinical status following treatment, we also use the 6-point ordinal clinical status severity scale. The remaining clinical outcomes include parameters which are easily measurable (need for O_2_ supplementation) or easily evaluable (need for antibiotic therapy for influenza complication, ICU admission, death). Influenza complications are retrieved from the discharge letter at the end of the hospital stay. The study nurses/physician collect the above data directly from the patients’ electronic medical records and enter it into the e-CRF. Clinical monitors will randomly check for data consistency and correctness.

Quality of life questionnaire EQ-5D-5L [17, 18] is filled out by the study nurses/physician on D0 and D90 post-treatment administration. At D90, the answers are collected by the study nurses/physician via the phone and are entered directly to the e-CRF. In case the patient is incapable of answering the questions, the questionnaire is not filled out.

#### Virology outcome measures

1. Viral shedding is measured by RT-PCR test and is expressed in CT values or copies/ml. Data is generated at the Laboratory of Virology of the Geneva University Hospitals and will be entered to the e-CRF by the study nurses/physician or directly by the biologist in charge or a trained laboratory technician.
2. Viability and infectiousness of the virus detected in the samples is measured by viral culture (expressed as a titer) at the Laboratory of Virology of the Geneva University Hospitals. Results will be entered to the e-CRF by the study nurses/physician or directly by the biologist in charge or a trained laboratory technician.

#### Safety outcomes

The incidence of serious adverse events unrelated to the natural course of influenza, serious adverse reactions, and suspected unexpected serious adverse reactions will be collected and reported following Swiss regulations. Reporting will be done by the sponsor investigator or by the co-investigators with the support of the CTU pharmacist.

#### Assessment of other outcomes of interest

Resistance mutations to baloxavir will be assessed by Sanger or whole genome sequencing. In case of Sanger sequencing, the partial sequence of 890–898 bp of the PA gene that we target includes all positions described to be associated with a decreased susceptibility (resistant) to baloxavir [19]. The screening and interpretation of the different mutations will be done through an algorithm co-developed between the Swiss national reference center of influenza and the Integrated database network system (Smartgene, Lausanne, Switzerland). The remaining respiratory samples, as well as the collected serum on admission and on discharge, will be conserved in a biobank located at the Laboratory of Virology of the Geneva University Hospitals.

### Plans to promote participant retention and complete follow-up {18b}

Subjects who withdraw or are withdrawn from the study prior to IMP administration will be replaced. No follow-up of these subjects will be performed, and no data will be analyzed. Subjects withdrawing or withdrawn after randomization and IMP administration will not be replaced. Data and samples collected before withdrawal will be analyzed. The reason for study discontinuation or deviation will be recorded in a specific section of the e-CRF.

Patients who drop out will be right censored at the time of withdrawal, with reasons for withdrawal documented. For deceased patients, clinical improvement will be considered as having never occurred until the end of follow-up: those patients will be right censored at 30 days.

### Data management {19}

Data management will be performed by the local Clinical Trial Unit (CTU) at the Geneva University Hospitals. The CTU is certified ISO 9001/2008, and the unit guarantees best practices in the field of clinical data management. Data are physically stored in secuTrial® using a dedicated CDMS software (Clinical Database Management System).

Data entry: To prevent data loss and preserve data integrity, all clinical and basic laboratory data will be directly entered into the e-CRF from the patients’ digital or paper-based medical records. All original data will be kept safely in the digital medical records system as well as in a dedicated, locked office with access only to study personnel. Virological data generated by the Laboratory of Virology at the Geneva University Hospitals will be entered into the e-CRF by the study nurse/physician. One hundred percent of primary outcome data will be checked by study monitors. Other data will be subject to regular random data consistency and correctness checks in the e-CRF by study monitors. Automatic value plausibility checks will be integrated into the e-CRF system.

Coding: All patient data will be coded in the e-CRF. Only personnel working closely on the project (PI, co-investigators, study nurses/physicians, study monitors) will have access to the uncoded data.

Data will remain accessible for authorized people throughout the study conduct and until the final publication of results.

Data security: Access to the patients’ digital medical records is controlled by the hospital in each recruiting center. Only authorized health care personnel can access the system. In our study, only the above-detailed study personnel will have access to the patients’ original digital and paper medical records. Access to the coded data on e-CRF is going to be strictly regulated as well. Only authorized personnel can enter or modify data; every data entry and modification will be automatically tracked.

Data storage: All data and applications are physically stored in dedicated data centers during 20 years on the Geneva University Hospitals’ premises.

For each set of data, quality control and triggers to computerized logic and/or consistency checks will be systematically applied to detect errors or omissions. After integration of all corrections in the complete set of data, the database will be locked and saved before being released for statistical analysis. Each step of this process will be monitored through the implementation of individual passwords and/or regular backups to maintain appropriate database access and to guarantee database integrity.

### Confidentiality {27}

The study investigator affirms and upholds the principle of the participant’s right to privacy and that they will comply with applicable privacy laws. Especially, the anonymity of the participants will be guaranteed when presenting the data at scientific meetings or publishing them in scientific journals.

Individual subject medical information obtained as a result of this study is considered confidential, and disclosure to third parties is prohibited. All patient data will be coded in the e-CRF.

Only personnel working closely on the project (PI, co-investigators, study nurses/physicians, study monitors) will have access to the uncoded data. Coding will be performed by the study team in each center. The identification key will be stored in a password-protected Excel file located on a secured server at each study site.

### Plans for collection, laboratory evaluation and storage of biological specimens for genetic or molecular analysis in this trial/future use {33}

Biological specimen collection is done by the study nurses and/or study physician, with sampling material provided by the sponsor. The use of coded labels corresponding to the randomization number upon biological specimen collection ensures patients’ privacy and specimen tracing. All biological specimens are handled and stored according to a dedicated standard operating procedure (SOP) at the respective hospital laboratories. Aliquots and leftover samples are stored at −80 °C at the sites and are sent at the end of each flu season to the Geneva University Hospitals’ Laboratory of Virology, where they are included in the study biobank before being analyzed in batch.

All leftover and serum samples taken for future research purposes will be stored for an indefinite duration (minimum 20 years) in the INFLUENT biobank located at the Geneva University Hospitals’ Laboratory of Virology.

Only coded samples from patients consenting will be used for future research purposes.

## Statistical methods

### Statistical methods for primary and secondary outcomes {20a}

The primary outcome (time to clinical improvement) is a time to event data. The “clinical improvement-free survival” will be estimated according to the Kaplan–Meier method and compared between groups (baloxavir vs. placebo) using a Cox model. The conditional effect of baloxavir on time to clinical improvement will be estimated with the following covariates included in the model (all collected at inclusion): age, vaccine status (yes/no), NEWS2 baseline score, BMI, and study site. As recommended by the EMA, the FDA, and ICH guidelines, we prospectively planned to include key prognostic factors as covariates in the statistical models, regardless of whether they were used for stratification during randomization. The purpose of this approach is to improve the precision of the estimated treatment effects and to account for potential imbalance in important baseline characteristics, even in randomized trials. Covariates that were not considered clinically relevant or did not demonstrate prognostic value based on prior knowledge were not included to avoid overfitting and preserve model parsimony.

First intention will be to include age and NEWS2 score as continuous variables. If the log-linearity assumption does not hold, age will be categorized as ≥65 vs. <65 years, and BMI (<30 vs. ≥30 kg/m^2^), and NEWS2 score as ≤7 vs. >7. This multivariable model will be considered the primary model. The study being a superiority trial, the principal analysis will be based on the intention-to-treat (ITT) population among all patients included in the trial independently of treatment timing POS. The same analysis will be done in the subgroup of patients receiving the study treatment ≤ 72 POS.

Clinical improvement-free survival will be estimated using the Kaplan–Meier method. For hypothesis testing, a Cox proportional hazards model will be employed. The hazard ratio (HR) will be estimated from the Cox model, and two-sided 95% confidence intervals will be derived using standard errors of the log (HR), calculated from the observed Fisher information matrix. The Breslow method will be used to handle tied event times. The proportional hazards assumption will be assessed visually using log(−log[survival]) plots. In case of a violation of this assumption, a secondary analysis will be conducted by incorporating time-dependent covariates to account for non-proportional effects.

#### Definition of the ITT population

As recommended by ICH E9 guidelines [20], the primary analysis will be conducted according to the intention-to-treat principle, using the full analysis set (FAS). The ITT includes all randomized patients, except those with critical protocol deviations that prevent any valid post-randomization analysis. Specifically, the following patients will be excluded:

- Those who did not meet key eligibility criteria (i.e., major inclusion/exclusion violations)
- Those who did not receive at least one dose of the investigational treatment
- Those with no post-randomization data available

This definition is consistent with the ITT approach, which aims to preserve the advantages of randomization while ensuring sufficient data are available for meaningful analysis.

#### Definition of relevant intercurrent events

The estimand of interest targets the effect of treatment on the time to clinical improvement within 30 days of randomization. The variable is defined as the time (in days) from randomization to clinical improvement, with a maximum follow-up of 30 days. Death before improvement is considered an intercurrent event and will be handled using a composite strategy, whereby death is interpreted as a failure to improve. In this case, the time to improvement will be right censored at day 30. Other intercurrent events are considered irrelevant to the definition of the treatment effect and will be addressed using a treatment policy strategy, meaning that patients will be analyzed according to their randomized treatment regardless of these events.

The treatment effect will be summarized by the adjusted hazard ratio with corresponding 95% confidence intervals.

#### Clinical secondary outcomes

Status severity score at day 7 will be compared between groups using a Cochran-Mantel-Haenszel test stratified on center.

Length of stay (from treatment administration to hospital discharge) and duration of O_2_ supplementation will be compared between groups using a linear regression model adjusted on center. The Huber-White method will be used to compute a robust variance estimator of the coefficients. Depending on the number of deceased patients, competitive risk analyses may be preferred to a linear regression model. Risk of in-hospital clinical failure will be compared between groups using a Cochran-Mantel-Haenszel test stratified on center. Risk difference and relative risk stratified on center will be estimated using the Mantel-Haenszel method.

Mortality and risk of influenza-related complications at day 90 will be compared between groups using a Cochran-Mantel-Haenszel test stratified on center. Risk difference and relative risk stratified on center will be estimated using the Mantel-Haenszel method.

Mean number of antibiotic days (AD) for respiratory illness will be compared between groups using a linear regression model adjusted on center.

Quality-of-life score at D90 will be compared between groups using a linear regression model adjusted for center and quality-of-life score at baseline.

#### Virology secondary outcomes

Proportion of patients with detectable RNA on day 3 post-treatment administration will be described using counts and percentages.

Proportion of patients with RNA detection and proportion of patients with an infectious viral load at day 3 post-treatment will be compared between groups using a Cochran-Mantel-Haenszel test stratified on center.

RNA and infectious viral load on day 3 (expressed in CT value and copies/ml and viral titer) will be compared between the two groups using a linear regression model adjusted for center and baseline viral load.

#### Safety secondary outcomes

SAEs unrelated to the natural course of a severe influenza infection, SAR, and SUSAR will only be described as count and/or percentage. As we do not expect them to happen, no comparison is planned between the 2 groups.

### Interim analyses {21b}

We will perform a blinded sample size re-estimation after the second influenza season. The objective will not be to evaluate the treatment effect, but to check the basis of our sample size calculation (median time to clinical improvement and prevalence of patients with symptoms evolving for less than 72 h). No other interim analysis is planned.

Early termination of the trial: Decision about early trial termination is the responsibility of the Safety Monitoring Board (SMB). If any safety signals suggest that withholding antiviral treatment could endanger participants, or if new research findings (from randomized, placebo-controlled trials in hospitalized adults) demonstrate the benefits of antiviral therapy in this population, the SMB will determine whether early termination of the study is necessary.

### Methods for additional analyses (e.g., subgroup analyses) {20b}

Subgroup analysis: using the same approach as in the primary analysis, the clinical efficacy of baloxavir in decreasing time to clinical improvement compared to placebo will be assessed in the following subgroups: patients with symptoms evolving for less than 72 h at treatment administration.

### Methods in analysis to handle protocol non-adherence and any statistical methods to handle missing data {20c}

As our primary outcome is based on data collected during regular care of hospitalized patients, we do not expect to have missing data.

### Plans to give access to the full protocol, participant-level data and statistical code {31c}

The full protocol is accessible on demand at any time upon request to the principal investigator. Datasets originating from our project and used in case of the publication of our results will be deposited in the institutional open-access data repository: “Dryad” and will be made public at the time of the publication. Metadata will be searchable and discoverable.

## Oversight and monitoring

### Composition of the coordinating center and trial steering committee {5d}

The composition of the coordinating center, roles and responsibilities: The coordinating PI who is also the sponsor representative and the trial coordinator who is also a Co-PI are managing the trial at the Geneva site as well as at all the other Swiss recruiting centers. There are 9 other Co-PIs at the coordinating site. Four of them are responsible for patient recruitment, others are responsible for the oversight of the sample analysis at the Geneva University Hospitals’ Virology Laboratory, others are responsible for the oversight of the study’s biobank, and others have support function due to their extensive experience in RCTs. The trial does not have a steering committee.

Trial guidance: At the coordinating center, the coordinating PI and the trial coordinator assume the day-to-day support for the trial. They meet with the local PIs and the study nurses at the three other Swiss centers weekly online to discuss and resolve any issues concerning patient inclusion, follow-up, safety measures, and data entry to the e-CRF. The study coordinator connects daily with the study nurses at the coordinating center for the same purposes as detailed above. Outside of flu season, the coordinating PI, the study coordinator, and the local PIs meet every second week to discuss questions concerning study design, regulatory authorities’ submissions, and other fundamental study issues.

Handling of the data management system, performing monitoring activities, and providing an electronic data capture solution (Secutrial database) for storage of participants’ CRF is done by the Clinical Trial Unit at the Geneva University Hospitals. In addition, statistical analysis is performed by the clinical trial unit (CTU) Geneva.

### Composition of the data monitoring committee, its role and reporting structure {21a}

The Safety Monitoring Committee (SMC) is constituted by 4 members, each being experts either in clinical trials or in the field of influenza and respiratory infectious diseases. One of the members is a statistician.

Task: Ensuring participant safety and detecting safety signals towards not giving antiviral is putting the patients in danger. The SMC first met before the start of the trial to discuss the protocol, the trial, and to have the opportunity to clarify any aspects. Further on, the SMC should meet once a year within 3 months after the end of each influenza season. The meetings and dates are set up by the sponsor in agreement with the SMC.

The Interim Data Report discussed after each influenza season includes only data containing potential safety signals: mortality, intensive care admission, and collected safety events according to the protocol. Data will remain blinded, with the lifting of the blind only at the request of the SMC.

After the second influenza season, on top of the data cited above, a sample size re-estimation will be performed to check the assumptions made on the median time to clinical improvement and the prevalence of patients with symptoms evolving for less than 72 h. There will not be an interim analysis, nor a futility analysis during the trial as their power would be insufficient. Depending on the data and for the sake of patients’ safety, the SMC may order additional analysis.

The SMC is independent of the sponsor, and its members do not have competing interests. A charter is available on request.

### Adverse event reporting and harms {22}

Baloxavir’s safety profile is well known and extremely favorable. Adverse events are not part of our study questions. Therefore, only serious adverse events considered unrelated to the natural history of influenza will be reported to the competent authorities in the annual safety report. SAR and SUSAR will be reported timely to the competent regulatory authorities.

### Frequency and plans for auditing trial conduct {23}

Monitoring is organized by the CTU Geneva. CTU Zurich and CTU EOC are involved in the monitoring of the Zurich and the Locarno-Lugano sites, respectively.

The monitoring strategy has been defined by the Clinical Trial Unit as low-risk, according to the SCTO guidelines and the Risk-Based Monitoring (RBM) Score Calculator published on https://www.sctoplatforms.ch/en/tools/risk-based-monitoring-score-calculator-31.html.

A site initiation visit was conducted before the inclusion of the first participant. During routine monitoring visits, the verifications concern: existence and completion of informed consent forms, eligibility criteria, primary outcome including the values necessary to calculate the last two NEWS2 scores before hospital discharge, influenza vaccination history, age, BMI, clinical status on day 7, investigational medicinal product dispensing and accountability, trial master file/investigator site file completeness, whenever necessary.

A close-out visit will be conducted at the end of the study. The study is considered as a low-risk trial; therefore, there is no Project Management Group, Trial Steering Group, or Data Monitoring Committee. Please find details about the Safety Monitoring Committee in paragraph 21a.

### Plans for communicating important protocol amendments to relevant parties (e.g., trial participants, ethical committees) {25}

Substantial amendments will only be implemented after approval of the competent ethics committee and the competent authority (Swissmedic), respectively. After approval by the competent authorities and before implementation, all substantial amendments are communicated to the funder. The revised protocol is then sent to the local PIs of all recruiting centers to be stored in the ISF. All substantial amendments will be communicated to participants of the study if they occurred during the length of their follow-up. A substantial amendment will also lead to an update of all trial registries (clinicaltrials.gov, NCTCP).

All non-substantial amendments will be communicated to the competent authority as soon as possible if applicable and to the competent ethics committee within the annual safety report. All non-substantial amendments are also communicated to the funder annually in the interim scientific report.

Protocol deviations are fully documented and collected in the dedicated deviation log in the trial master file as well as in the ISFs.

### Dissemination plans {31a}

Results will be published in a peer-reviewed open-access journal as an original article. The manuscript will be written by the PI and the project manager with the help of the study team. All authors will critically review the manuscript. We do not foresee the use of professional writers. We will grant public access to the full study protocol. Datasets originating from our project and used in case of the publication of our results will be deposited in the institutional open-access data repository: “Dryad” and will be made public at the time of the publication. Metadata will be searchable and discoverable.

Research results will also be communicated to the general public. We foresee research result publication in laymen’s terms during the year following the end of the study. We are going to involve patient partners in formulating a lay summary of the research results. We plan to publish in the hospital’s information magazine as well as in the form of a press release with the help of the HUG’s communication department.

#### Patient public involvement

Dedicated patient partners participated in the design of the study, in the discussion about the ethical aspects of using placebo as a comparator to baloxavir, in the design of the protocol, the ICF, and the advertisement material (flyers). They actively participated in the reflection about patient-centered outcomes and what type of tool would be the most suitable and least burdensome to measure these outcomes. They also participate after each influenza season in the discussion about challenges in patient recruitment and how to address them. Patient partners will be involved at the end of the trial in results dissemination destined for lay people.

## Discussion

Trial procedures are documented in the “SOPs.” Such SOPs cover (are not limited to) participant screening, inclusion, randomization, follow-up, safety, sample collection, and laboratory processing. Regular weekly meetings will be held with all centers to discuss inclusion, pitfalls, and difficulties. The coordinating PI and the study coordinator will, in addition, be available every day in case of question relative to study inclusion or participant safety.

## Trial status

The first version of the protocol restricted the inclusion in the trial to participants with symptoms ongoing for more than 48 h. With the updated WHO guidance on influenza management published on September 12, 2024, an amendment was successfully submitted to the competent authorities to be able to include patients independently of the duration of their symptoms. Version 2.0 of the protocol dated October 29, 2024, was accepted by the competent ethics committee on November 6, 2024, and by Swissmedic, the competent authority, on November 21, 2024. Study recruitment opened December 1, 2024. The first participant was included on December 13, 2024; recruitment is planned to be completed after the 2026/2027 influenza season, with an end of patient follow-up in July 2027.

## Supplementary Information

Additional file 1. SPIRIT checklist

## Data Availability

The final dataset will be accessible by the PI and the trial statistician. The final dataset used in case of the publication of the results will be deposited in the institutional open-access data repository: “Dryad” and will be made public at the time of the publication. Remaining biological samples of participants who agreed will be deposited in the INFLUENT corresponding biobank registered on the Swiss biobanking platform. Further use of the samples will be decided by the biobank steering committee, as regulated by the biobank regulation document.

## Abbreviations

AD: Antibiotic days
AE: Adverse event
ARDS: Acute respiratory distress syndrome
ASR: Annual safety report
BASEC: Business Administration System for Ethical Committees (https://submissions.swissethics.ch/en/)
BMI: Body mass index
CA: Competent authority (e.g., Swissmedic)
CDMS: Clinical Database Management System
CEC: Competent Ethics Committee
CRF: Case report form
ClinO: Ordinance on Clinical Trials in Human Research
eCRF: Electronic case report form
CTCAE: Common terminology criteria for adverse events
CTU: Clinical trial unit
D: Day
DSUR: Development safety update report
GCP: Good Clinical Practice
HIV: Human immunodeficiency virus
Ho: Null hypothesis
HRA: Federal Act on Research involving Human Beings
ICU: Intensive care unit
IMCU: Intermediate care unit
IMP: Investigational medicinal product
IIT: Investigator-initiated trial
ITT: Intention to treat
NAI: Neuraminidase inhibitor
NEWS2: National Early Warning Score 2
NPS: Nasopharyngeal swab
OR: Odds ratio
PI: Principal investigator
PO: Per os
POS: Post onset of symptoms
RBM: Risk-Based Monitoring
RT-PCR: Reverse-transcription polymerase chain reaction
SAE: Serious adverse event
SAR: Serious adverse reaction
SDV: Source data verification
SMC: Safety Monitoring Committee
SOP: Standard operating procedure
SPC: Summary of product characteristics
SUSAR: Suspected unexpected serious adverse reaction
TMF: Trial master file
